# Prime Time for Artificial Intelligence in Interventional Radiology

**DOI:** 10.1007/s00270-021-03044-4

**Published:** 2022-01-14

**Authors:** Jarrel Seah, Tom Boeken, Marc Sapoval, Gerard S. Goh

**Affiliations:** 1 Department of Radiology, Alfred Health, Melbourne, VIC Australia; 2 Department of Neuroscience, Monash University, Melbourne, VIC Australia; 3 Vascular and Oncological Interventional Radiology, University of Paris, Hopital Européen Georges Pompidou, Paris, France; 4 Department of Surgery, Central Clinical School, Monash University, Melbourne, VIC Australia; 5 National Trauma Research Institute, Central Clinical School, Monash University, Melbourne, VIC Australia

**Keywords:** Machine learning, Interventional radiology, Deep learning, Artificial intelligence, AI

## Abstract

Machine learning techniques, also known as artificial intelligence (AI), is about to dramatically change workflow and diagnostic capabilities in diagnostic radiology. The interest in AI in Interventional Radiology is rapidly gathering pace. With this early interest in AI in procedural medicine, IR could lead the way to AI research and clinical applications for all interventional medical fields. This review will address an overview of machine learning, radiomics and AI in the field of interventional radiology, enumerating the possible applications of such techniques, while also describing techniques to overcome the challenge of limited data when applying these techniques in interventional radiology. Lastly, this review will address common errors in research in this field and suggest pathways for those interested in learning and becoming involved about AI.

## Introduction

Artificial intelligence (AI) has been prominent in different fields including diagnostic radiology. AI breakthroughs are now also empowering the field of interventional radiology (IR) and recent surge in popularity was initially driven by the phenomenal success of deep neural networks in processing unstructured data such as images and audio through pattern recognition. The term AI has come to encompass all forms of machine learning (ML). In this review the term AI is used synonymously with ML, referring to techniques that construct predictive models from data, including deep learning, radiomics and other traditional machine learning techniques. The importance of the usage of AI is summarized in (Table 1).

**Table 1:** The importance of artificial intelligence

Has the ability to incorporate and analyse a large amount of complex data rapidly
Identifies trends and patterns only partly detectable by humans
Independent of human bias in decision-making
Has the potential to aid interventional radiologists in the diagnosis, treatment and follow-up of disease

A brief history of the field is difficult to summarize given its fragmented nature; however, the most popular techniques currently, artificial neural networks, date back to work by Rosenblatt[1] on the concept of the perceptron in the 1960s.

Artificial neural networks are inspired by the connectionist design of biological neural networks[2], comprising of artificial “neurons” which receive input from other neurons or the environment, performing a nonlinear activation function[3] on the sum of this input, and passing its output to other neurons. In short, each layer in an artificial neural network is a mathematical model loosely mimicking biological neurons, receiving information from one or more sources, processing the information and producing a response. This information is passed to other neurons for further analysis. To train a neural network, a large dataset of paired inputs and desired outputs, referred to as labels, must be collected. The training process then refers to altering the parameters (typically the weights) for each neuron such that the network produces the desired outputs for each input. Currently, the most popular architecture for processing images is the convolutional neural network (CNN) [4], where neurons are organized spatially, taking inputs from adjacent pixels and processing it into higher-order representations before passing this information onto successive layers, eventually producing predictions. These predictions are compared to the labels, and the gradient of the parameters of the network, with respect to the error, is calculated through a process referred to as backpropagation[5]. Supervised learning is a type of ML that utilizes a set of input and output labelled training data [6] and can be used to estimate relationships between input and output parameters.

AI using artificial neural networks were recently re-popularized in 2012 by Krizhevsky et al. [7] with the development of large-scale parallel processing through graphics processing units combined with the availability of large datasets. In their revolutionary publication in 2012, Krizhevsky et al. [7] trained a deep CNN to classify over 1 million images (from the ImageNet contest) into 1000 different classes. Their network was built with 650,000 neurons and 60 million parameters. Though networks have since become even more complex, the AlexNet model created by Krizhevsky remains a reference for image classification. It is important to acknowledge that CNNs are still at the core of most research in this field at this nascent stage.

Recent research in medical imaging AI has focused on 3D CNNs such as 3D versions of popular 2D CNNs such as EfficientNet or Densenet. [8] Alternative techniques such as 2.5D networks (taking into account axial, coronal and sagittal planes) have also been developed. [9]. Even more recently in the wider AI space is the shift to purely attention-based mechanisms such as vision transformers [10] even for image-based tasks. These popular architectures are mentioned in brief as it is beyond the scope of this review article to describe these in detail. These approaches work remarkably well when networks are scaled up to millions or billions of parameters. Where traditional ML algorithms such as logistic regression tend to overfit with so many parameters, neural networks remarkably appear to work better with more parameters, particularly when multiple layers are stacked deeply. [11] Why this is the case remains one of the central mysteries of deep learning.

One limitation of neural networks is that most deep learning algorithms have been restricted to data-rich domains such as photography or speech recognition, as training these algorithms requires large datasets. Radiology is well suited to this, being a data-rich specialty born into the information age, with explosive growth in AI research in diagnostic radiology[12]. Interventional radiology when compared to diagnostic radiology deals with much smaller datasets and may seem much less appealing to the AI researcher.

In comparison with other interventional and procedural fields such as surgery or endoscopy, IR is data-rich. IR is one of a few specialties where a record is kept of the entire procedure in a standardized format, is available retrospectively and these datasets are mostly unexploited today. With the recent developments in few shot learning made by the deep learning community[13], novel techniques may drastically reduce the dataset size required for clinically effective algorithms, making it truly the prime time for AI in interventional radiology.

## Applications

Potential applications of AI in IR can be divided into pre-procedural, peri-procedural and post procedural.

### Pre-procedural Setting: Improving Patient Selection

Better patient selection is similar to the concept of precision medicine. AI decision support systems may help tailor treatment decisions based on imaging phenotypes, yielding better clinical results.

Interventional radiologists often rely on multidisciplinary boards for oncological treatment strategies. These board discussions perform multiparametric risk-stratification, integrating the patient’s full data before a treatment is advised.

Several AI applications replicate and outperform these discussions by predicting the outcome from data available in each specialty (radiology, histology, molecular biology, etc.). The ability to incorporate clinical information, radiomics and genetic information may improve the objectivity and accuracy of decision-making. Such an approach could potentially play a role in triaging patients for IR and subsequent therapy by assessing risks and making predictions about therapeutic outcomes [14]

An example of this application in the field of acute ischaemic stroke is the use of CT perfusion software in endovascular clot retrieval to estimate physiological parameters such as ischaemic core and penumbral volume, facilitating the selection of patients who are likely to have an optimal outcome. AI approaches to identifying patients for clot retrieval are being investigated, for instance based on CT angiography rather than CT perfusion. [15–17]

Pretreatment patient selection using deep learning is also being investigated for interventional oncology. Morshid et al. [18] describe an algorithm to predict response to transcatheter arterial chemoembolization for hepatocellular carcinoma (HCC) using pretreatment CT, combined with the clinical BCLC stage. They demonstrate that an AI model utilizing image and clinical features can outperform traditional staging systems in predicting benefit from TACE. Similarly, Peng et al.[19] developed a deep learning model that predicts response to TACE with an accuracy of 84%. Kim et al. [20] demonstrated that a combined radiomics and clinical model of HCC in response to TACE improved survival estimation when compared to clinical models alone. Other research in HCC treatment has found similar results when applied to surgical or thermo-ablative resection [21].

Multimodal planning may also integrate genetic information using AI models. Ziv et al. [22] trained a model to identify the genes most predictive of response to TACE and Kuo et al. [23] utilized radiomic analysis to identity imaging phenotypes associated with doxorubicin drug response gene expression in HCC.

### Peri-procedural: Improving Procedures

AI can improve interventional procedures by accelerating computationally intensive or manual procedures, such as the correction of translational motion via pixel shifting in angiography. Traditional image registration techniques such as those proposed by Meijering et al. [24] are computationally intensive and have not had widespread uptake. Deep learning approaches may speed up corrected digital subtraction angiography, such as methods proposed by Gao et al. [25] which use generative adversarial networks to generate subtraction images without the preliminary non-contrast acquisition, avoiding the issue of translational motion entirely. This is achieved by acquiring a dataset of satisfactorily subtracted images paired with the unsubtracted images and training a neural network to predict the subtracted images from the unsubtracted image. This teaches the neural network anatomical and physical assumptions about the nature of angiographic contrast. The resultant neural network is capable of predicting the subtracted images from the unsubtracted angiographic images, without the use of the preliminary non-contrast acquisition.

Deep learning approaches have also been applied to identifying guidewire and catheters during angiography [26]. Such methods may permit more advanced algorithms such as virtual road mapping of the vasculature without contrast. Real-time AI registration algorithms could superimpose high-resolution preoperative imaging with procedural fluoroscopy, guiding the interventional radiologist during catheter manipulation.

AI-based ultrasound guidance [27] has been used in echocardiography to help guide the acquisition of echocardiograms. Deep learning algorithms estimate diagnostic quality of the image and suggest manoeuvres to improve the quality of such images. AI may provide recommendations on needle trajectory or other facets of interventional procedures, which may be particularly useful for novice operators.

The selection and personalization of endovascular devices is another area for AI. Yang et al. [28] used AI to segment and quantify stenosis on coronary angiography. Such algorithms could be used to objectively select the optimal stent for each lesion. Lee et al. [29] imagine a future where AI may guide the personalized 3D printing of cardiovascular stents.

Cho et al.[30] developed AI to predict fractional flow reserve of coronary lesions on angiography. This opens the way to extract hemodynamic parameters/physiological parameters from angiography, and AI may be able to even estimate flow distribution maps in the future.

AI has been proposed for skin dose estimation by taking into account angulation of the X-ray tube and tissue density. Radiation exposure during endoscopy has been reduced using an AI-equipped fluoroscopy unit with an ultrafast collimation system that reduced radiation exposure by ~ 38%. Similar techniques could be used in interventional radiology. [31]

Respiratory motion compensation in PET/CT imaging has been implemented via elastic motion correction algorithms where AI determines a blurring kernel between a single motion corrected image and a single non-motion corrected target image. This results in a final image with reduced motion[32]. Similar applications could be applied in live fluoroscopy.

### After Treatment: Improving Follow-Up

Following treatment, AI has a role to play in measuring response to treatment, prognostication and determining future management.

Most criteria used in diagnostic radiology for treatment response were not developed for interventional radiology, which may lead to misevaluation during follow-up. AI research could help better assess these specific treatment responses. AI can be useful in longitudinal studies during follow-up of treatments to detect subtle changes between images identifying disease progress or recurrence earlier.

In oncology, automated volumetric measurements of tumour sizes or response evaluation criteria in solid tumours (RECIST) reads may be possible through deep learning. [33]. RECIST criteria themselves as a marker of response can be outperformed by AI. Dohan et al. [34] developed a radiomic signature that was able to predict overall survival and identify good responders better than RECIST1.1 criteria in patients with liver metastases from colorectal cancer treated with chemotherapy. The same models could be applied to IR treatment in liver metastases, potentially outperforming routine RECIST and equivalent criteria.

Procedural findings and histological features can also play a role in the choice of adjuvant therapy as suggested by Saillard et al.[35] who built a prediction model of survival after HCC resection based on pretherapeutic and histological preprocedural features.

Similarly, AI has a role in assessing response to treatment in acute ischaemic stroke. Thrombolysis in cerebral infarction (TICI) scores are often used to grade results following endovascular clot retrieval. AI can improve interobserver reliability and thereby improve the utility of such scores in prognosticating patients. [36, 37] AI algorithms may help reduce the time required to interpret post-treatment imaging and improve inter-observer variability. The ability of AI to extract quantitative metrics holds the promise of personalizing management plans, particularly in complex chronic conditions such as cancer.

While genetics and molecular pathology have played a large role in precision medicine, pre- and post-treatment imaging may identify additional disease phenotypes as well as quantify intervention success, which may help fine-tune management by prognosticating as well as determining the timing and need for follow-up imaging. [38]

## Practical Challenges

The breadth of potential applications of AI in interventional radiology has seen a rise in academic papers published on this subject. Such projects face a common set of challenges..

The major challenge facing AI in interventional radiology is the relatively small dataset sizes when compared to diagnostic radiology, or in fact, to other non-medical applications of AI entirely. For instance, ImageNet, a widely used natural imagery database, contains over 14 million images. [39] In contrast, most medical applications have dataset sizes in the hundreds to thousands of unique samples. Therefore, standard deep learning models are difficult to train from scratch.

Perhaps the most simple method to reduce the number of samples required for a useful model is transfer learning, where models trained on different datasets might be used as a starting point, as information that these models might have learned from other datasets may be translated to this setting as well. [40]

Other approaches include the use of handcrafted features, an approach popular in the “traditional” computer vision literature in the early 2000s. These approaches, also known as radiomics when applied to imaging, reduce the number of required samples as the model does not have to learn the low-level features itself.

Another approach is to use data augmentation – standard transformations like affine transforms, adjusting brightness and contrast are useful, but novel augmentation techniques like MixUp [41] may help researchers get more out of their data. Medical imaging is often acquired quite differently from natural imagery, through techniques such as tomographic reconstruction. This offers the opportunity for different types of augmentations to introduce artefacts which are more typical in this setting, such as physics-based data augmentation [42]. Although this technique was found to be unsuccessful in previous work, it may be prove to be useful in more challenging datasets.

Recent developments in the deep learning field in semi-supervised learning, such as few-shot and zero-shot learning techniques [13, 43–46], may also help reduce the number of labelled samples required, by using unsupervised datasets as additional information.

For categorical data, such as models using clinical variables like age and sex, oversampling techniques such as synthetic minority oversampling technique (SMOTE) [47] may help generate synthetic data points that may improve an AI model’s performance.

Small dataset sizes also exacerbate common mistakes made in AI projects. The use of checklists [48] may help prevent some of these avoidable errors. Common errors include the failure to split data by patient – i.e. including studies from a single patient in both the training and test datasets. This may lead to the model memorizing patient specific features, leading to over-optimistic results that do not translate into clinical practice. Other errors include not fully describing the hyper-parameter optimization process, or optimizing the hyper-parameters on the testing set, which again leads to over-estimation of the model’s performance.

The training and testing datasets must also have defined inclusion and exclusion criteria to prevent “Frankenstein” datasets[48], where positive and negative cases are drawn from different sources, potentially leading to data leakage as the AI model may recognize features specific to the dataset source rather than the disease of interest.

To prevent overstating the significance of any result, especially in small datasets, any measure of performance such as the accuracy or the area under the receiver operating characteristic (AUC) should be accompanied with confidence intervals. When applicable, AI models trained on images should be compared to a baseline clinical model using age, sex and other clinical features. If an AI model uses both imaging and clinical features, a sensitivity analysis should be performed by systematically modifying each input to assess its contribution on the final prediction. [49] In post-treatment and prognostication models, lack of complete follow-up in all participants is common and should be accounted for when measuring the accuracy of such models through censoring. [50]

Another source of unreliability stems from the constant evolution of clinical practice over time due to the introduction of new treatment approaches, technologies or changes in patient population [51]. Interventional radiology, in particular, is a rapidly evolving specialty with novel equipment and procedures constantly developing over time.

The use of AI in augmenting interventional radiologists is likely to increase as research in pretreatment, intra-treatment and post-treatment applications translate into clinical practice. Due to the potential benefits and risks for patients, stringent prospective evaluation such as controlled trials should be undertaken where necessary to ensure that promising applications translate well.

## How to Get Started in Ai

Given the promise of AI in interventional radiology many clinicians may wish to get involved in AI research and development. Key factors to be able to successfully translate a project into clinical practice include a clear understanding of the clinical benefits andadvantages of using AI, the availability of data measured in independent samples (typically at a patient level), the use of computing resources such as graphics processing units or tensor processing units and the technical skills to construct an AI model.

Specific steps around training and coding of AI models are beyond the scope of this review article; however, it is becoming easier the advent of open source deep learning framework libraries such as Pytorch [52] and Tensorflow [53]. A recommendation for interventional radiologists who are interested in learning more about AI is to begin with learning basic software and data carpentry skills in programming languages such as Python [54] and then expand knowledge by undertaking courses in frameworks such as Pytorch and Tensorflow.

## Conclusion

The emergence of novel deep learning techniques and applications in interventional radiology is hugely exciting and offers multiple opportunities to aid in patient selection for intervention, improve patient care during interventional treatment and optimize post-treatment clinical follow-up. Interventional radiology with its smaller dataset sizes compared to diagnostic radiology stands to benefit from novel techniques such as semi-supervised learning, zero and few shot learning in the deep learning literature. The application of such techniques in interventional radiology must be rigorous and generalizable, and common errors must be avoided in order for successful clinical translation.
